# Management of chronic myeloid leukemia presenting with isolated thrombocytosis and complex Philadelphia chromosome: A case report

**DOI:** 10.1097/MD.0000000000027134

**Published:** 2021-09-03

**Authors:** Lu Gao, Ming-qiang Ren, Zu-guo Tian, Zhi-yuan Peng, Genghui Shi, Zhong Yuan

**Affiliations:** Department of Hematology, Affiliated Hospital of Zunyi Medical University, Zunyi, Guizhou Province, China.

**Keywords:** BCR-ABL, case report, chromosome translocation, chronic myelogenous leukemia, imatinib

## Abstract

**Rationale:**

Chronic myelogenous leukemia (CML) with thrombocytosis and complex chromosomal translocation is extremely rare in clinical setting. Here, we reported the clinical and pathological characteristics of CML patients, which were characterized by thrombocytosis and complex Philadelphia chromosome translocation. Moreover, we also introduced our therapeutic schedule for this patient as well as review relative literature.

**Patient concerns:**

A 24-year-old female presented with night sweating, fatigue, and intermittent fever for 1 month.

**Diagnosis:**

Fluorescence in situ hybridization results revealed that breakpoint cluster region (BCR)-Abelson (ABL) gene fusion in 62% of the cells and karyotyping showed a complex 3-way 46, XY, t(9;22;11) (q34;q11;q13) [19/20] translocation. This patient was diagnosed with CML complicated with thrombocytosis and complex Philadelphia chromosome translocation.

**Interventions:**

The patients received continuously oral imatinib mesylate tablets (400 mg) once a day.

**Outcomes:**

After treatment with imatinib for 3 months, the BCR/ABL^IS^ was less than 0.1% and achieved major molecular response. Moreover, the BCR/ABL^IS^ of this patient achieved major molecular response. The BCR/ABL^IS^ values at 6 months and 12 months were less than 0.01% and 0.0032%, respectively. And no BCR/ABL fusion was detected in the next 2 years follow-up period.

**Lessons:**

Imatinib might represent a preferred therapeutic option for CML patients with rare thrombocytosis and complex chromosomal translocation. In addition, BCR/ABL fusion gene examination in patients with thrombocytosis might represent an effective strategy to avoid the misdiagnosis of this specific CML population.

## 1 Introduction

Chronic myeloid leukemia (CML) is myeloproliferative neoplasm with an incidence rate of 1 to 2 cases per 100,000 adults. It is characterized by the presence of the Philadelphia (Ph) translocation-t (9; 22) (q34; q11), which results in breakpoint cluster region (BCR)/ABL in 5% to 8% of CML cases.^[1,2]^ CML patients frequently present with leukocytosis, although isolated thrombocytosis is rare. We herein report an extremely rare case of CML presenting with isolated thrombocytosis and the chromosomal translocation (9; 22; 11) (q34; q11; q13).

## 2 Case presentation

A 24-year-old female was admitted for night sweats, fatigue, and fever. Initial physical examination results showed that no obvious splenomegaly and abdominal distension was observed. Bone marrow immunophenotyping analysis in December 2018 revealed that platelet (PLT) (2100 × 109/L, normal range 100–300 × 109/L) count was significantly elevated and patient was initially misdiagnosed as essential thrombocytosis in other hospital. Despite hydroxyurea intervention could effectively reduce platelet count to 200 × 10^9^/L, it rapidly increased to 2300 × 10^9^/L within 1 week after drug withdrawal. This patient therefore was transferred to our center for further diagnosis and treatment in January 2019.

Routine blood test indicated normal: white blood cells 4.3 × 10^9^/L (normal range 4–10 × 10^9^/L), red blood cells 4.1 × 10^12^/L (normal range 3.5–5.5 × 10^12^/L), and Hb 133 g/L (normal range 110–150 g/L), and aberrantly high, platelet count of 2300 × 10^9^/L (normal range 100–300 × 10^9^/L).

**Figure 1:**
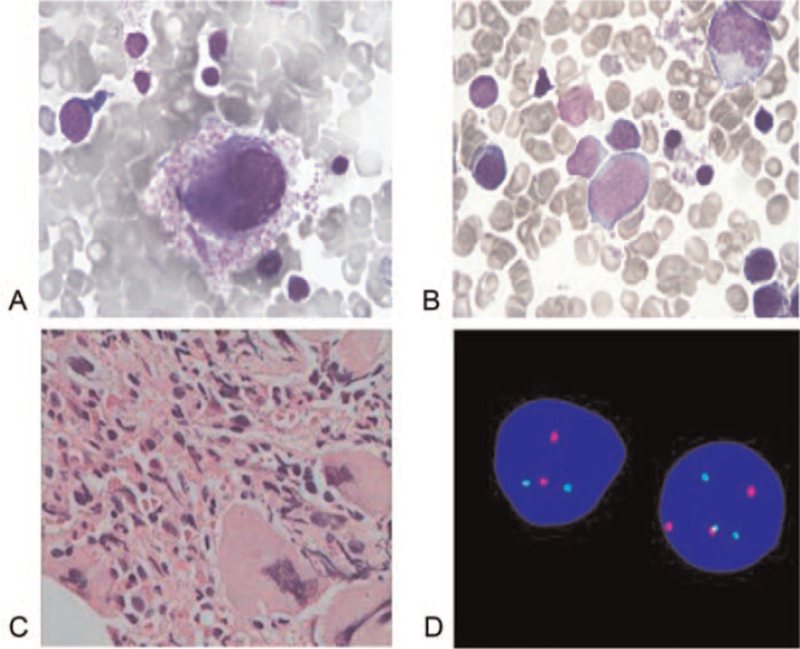
A, B, Representative images of bone marrow smears showing increased platelet count (10 × 100). C, Representative image of bone marrow biopsy showing megakaryocyte hyperplasia with large cells and segmented nuclei (10 × 10). D, Representative image of FISH showing green (BCR) signal on chromosome 22, orange (ABL) signal on chromosome 9, and yellow fusion signal (BCR-ABL) on derivative chromosome 9. FISH = fluorescence in situ hybridization.

**Figure 2:**
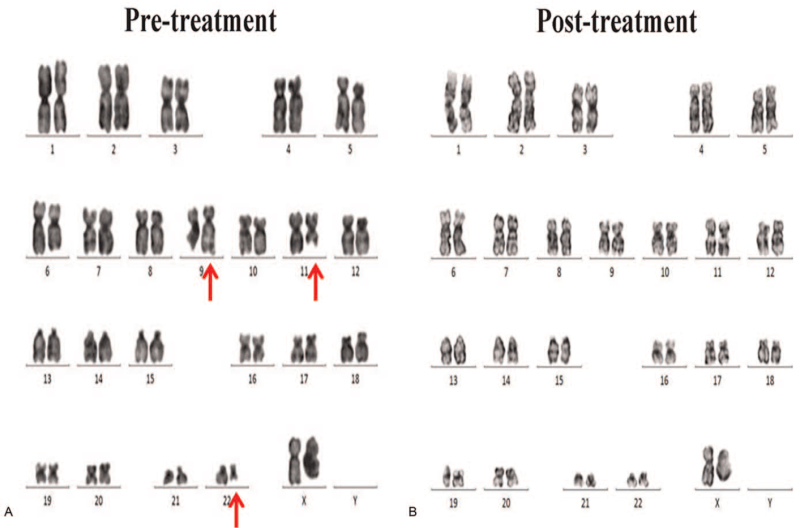
A, Karyotype showing a complex, 3-way chromosomal translocation: 46, XY, t(9;22;11)(q34;q11;q13)[19/20]. B, Normal karyotype: 46, XY [20/20].

Bone marrow aspirate smears showed obvious hypercellularity with a decreased myeloid to erythroid ratio of 0.61:1, as well as a decreased band-to-segmented neutrophil ratio. There were 286 megakaryocytes in the aspirate smear, and an increased number of platelets (Fig. 1A, B). Histological examination of bone marrow biopsy showed myeloid hyperplasia, with predominance of myelocytes and, metamyelocytes in the later stage. Erythroid hyperplasia and megakaryocytic hyperplasia (1–13 cells/HP) were also observed, and the megakaryocytes were large and had segmented nuclei (Fig. 1C). Genetic analysis results indicated that no obvious mutations on JAK-2 V617F, CALR exon 9, and MPL^W515L/K^ were observed. Fluorescence in situ hybridization revealed the BCR-ABL fusion in, 62% of the cells (Fig. 1D), and karyotyping showed a complex, 3-way 46, XY, t(9;22;11) (q34;q11;q13) [19/20] translocation (Fig. 2A). Based on all these results, the patient was diagnosed with chronic CML and treated with imatinib mesylate tablets (Gleevec) 400 mg orally once a day. Three months after diagnosis, no BCR-ABL fusion was detected and the karyotype was normal (Fig. 2B). Imatinib mesylate administration effectively reduced the PLT level as the time elapsed and maintained at normal level. Moreover, the white blood cells counts exhibited slight decrease after treatment, due to the hematologic toxicity of imatinib. Besides, the Hb level was found to have no obvious abnormity. The patient achieved the best treatment response 3 months after diagnosis with IS BCR-ABL1/ABL1 at 0.034%, which further decreased to 0.016% at 6 months and was undetectable at 12 months (Table 1). Written informed consent was obtained from the patient for publication of this case report. This study protocol was approved by the Ethics Committee of Zunyi Medical University.

**Table 1:** The therapeutic effect of imatinib on CML.

Date	Therapeutic dose	IS BCR/ABL (%)
Jan 18, 2019	Gleevec, 400 mg, po	92.41
Apr 26, 2019	Gleevec, 400 mg, po	0.034
Jul 15, 2019	Gleevec, 400 mg, po	0.016
Nov 1, 2019	Gleevec, 400 mg, po	0.002
May 21, 2020	Gleevec, 400 mg, po	<0.002

CML = chronic myeloid leukemia.

## 3 Discussion

To the best of our knowledge, this is the first report about an extremely rare case of CML presenting with isolated thrombocytosis and 3-way complicated Philadelphia chromosomal translocation. It's well-known that the typical clinical characteristics of CML are leukocytosis and splenomegaly, but rarely presented with isolated thrombocytosis.^[3]^ Findakly and Arslan^[4]^ reported in 20 CML patients with average age 40.5 years at diagnosis and male-to-female ratio of 7:13. The average platelet count of this cohort was 1923 × 10^9^/L (range: 584–8, 688 × 10^9^/L), and 2 of 15 patients who underwent physical examination or imaging exhibited splenomegaly.

CML is characterized by the genetic translocation, t (9; 22) (q34; q11.2), that results in the fusion of Abelson gene (ABL1) from chromosome 9q34 with the breakpoint cluster region (BCR) gene on chromosome 22q11.2.^[5,6]^ The Philadelphia chromosome translocation is observed in 90% to 95% of CML patients whereas more, complex translocations, involving a third chromosome are present in only 5% to 8% of the patients.^[7,8]^ Although variant translocations might involve any chromosome, the distribution of the breakpoints is nonrandom and, usually seen at some specific chromosomal bands, including 1p36, 2p22,3p21, 4q25,5q31, 6p21, 9q22, 10q22, 11q13, 12p13, 16p13,17p13,17q21, 17q25, 19q13, 21q22, 22q12, and 22q13.^[9–16]^ Additionally, Al-Achkar et al^[17]^ also reported a novel Ph chromosome-positive CML case lacking the BCR/ABL fusion gene on der(9) and instead harboring a new complex rearrangement formed by chromosomes 11 and 20, as well as 9 and 22. Moreover, Asif et al^[18]^ demonstrated a novel 4-way complex variant translocation involving chromosome 46,XY,t(4;9;19;22)(q25:q34;p13.3;q11.2) in a chronic myeloid leukemia patient.

Our patient exhibited solitary thrombocythemia without splenomegaly, and was initially misdiagnosed as essential thrombocythemia since genetic testing was not performed. Therefore, routine detection of the BCR/ABL fusion genes in peripheral blood and bone marrow smears is recommended for patients with unexplained solitary thrombocythemia to confirm the possibility of atypical CML, which is associated with poor prognosis and a median overall survival of 24 months.^[19]^ Valencia et al^[20]^ reported that imatinib effectively improved the prognosis of patients with variant Philadelphia chromosome similar to that of patients with the classic Philadelphia translocation. Therefore, we initiated treatment with daily imatinib 400 mg, which achieved best response in 3 months, and BCR/ABL negative status after 12 months.

In conclusion, imatinib resulted in a favorable outcome in an extremely rare case of CML presenting with isolated thrombocytosis and 3-way complicated Philadelphia chromosomal translocation t (9;22;11) (q34;q11;q13). Moreover, patients with isolated thrombocytosis should be routinely examined for BCR/ABL fusion gene, in order to effectively avoid the misdiagnosis of this specific CML population.

## Acknowledgment

The authors thank the patient for his approval to publication

## Author contributions

**Conceptualization:** Lu Gao, Zhong Yuan.

**Data curation:** Ming-qiang Ren, Zhi-yuan Peng.

**Formal analysis:** Zu-guo Tian.

**Funding acquisition:** Lu Gao.

**Investigation:** Zu-guo Tian, Zhi-yuan Peng, Genghui Shi.

**Methodology:** Genghui Shi.

**Writing – original draft:** Lu Gao, Ming-qiang Ren.

**Writing – review & editing:** Lu Gao, Zhong Yuan.
